# Nitrogen-containing bisphosphonate induces enhancement of OPG expression and inhibition of RANKL expression via inhibition of farnesyl pyrophosphate synthase to inhibit the osteogenic differentiation and calcification in vascular smooth muscle cells

**DOI:** 10.1186/s12872-024-04048-x

**Published:** 2024-09-17

**Authors:** Wei Xu, Lifeng Gong, Weigang Tang, Guoyuan Lu

**Affiliations:** 1 Department of Nephrology, First Affiliated Hospital of Soochow University, Suzhou, 215006 Jiangsu China; 2 Jiangsu Key Laboratory of New Drug Research and Clinical Pharmacy, Xuzhou Medical University, Xuzhou, 221004 Jiangsu China; 3 Department of Nephrology, Wujin Hospital Affiliated with Jiangsu University, Changzhou, 213000 Jiangsu China; 4 Department of Nephrology, People’s Hospital of Hainan Tibetan Autonomous Prefecture, Hainan Tibetan Autonomous Prefecture, Qinghai, 813099 China

**Keywords:** Nitrogen-containing bisphosphonate, Vascular smooth muscle cells, Vascular calcification, OPG, RANKL, Farnesyl pyrophosphate synthase

## Abstract

**Background:**

Nitrogen-containing bisphosphonate(N-BP)had been found to inhibit the osteogenic differentiation and calcification in vascular smooth muscle cells (VSMCs), but the mechanism is not clear. We intend to verify that N-BP induces enhancement of OPG expression and inhibition of RANKL expression via inhibition of farnesyl pyrophosphate synthase(FPPS) to inhibit the osteogenic differentiation and calcification in VSMCs.

**Methods:**

β-glycerophosphate (β-GP) was used to induce the osteogenic differentiation and calcification in VSMCs. VSMCs were treated with N-BP or pretreated with downstream products of farnesyl pyrophosphate synthase(FPPS) in mevalonate pathway, such as farnesol (FOH) or geranylgeraniol (GGOH). Alizarin red S staining and determination of calcium content were used to detect calcium deposition.Western Blotting were used to detect expressions of proteins(OPG and RANKL ) and osteogenic marker proteins (Runx2 and OPN).

**Results:**

β-GP induced the osteogenic differentiation and calcification in VSMCs, increased RANKL protein expression and had no significant effect on OPG protein expression. With the treatment of N-BP, the expression of OPG protein was increased and expression of RANKL protein was decreased in VSMCs undergoing osteogenic differentiation and calcification. In addition, N-BP reduced the osteogenic marker proteins (Runx2 and OPN) expression and calcium deposition in VSMCs undergoing osteogenic differentiation and calcification. These effects of N-BP on the osteogenic differentiation and calcification in VSMCs were concentration-dependent, which could be reversed by the downstream products of FPPS, such as FOH or GGOH.

**Conclusion:**

N-BP increases OPG expression and decreases RANKL expression via inhibition of FPPS to inhibit the osteogenic differentiation and calcification in VSMCs.

**Supplementary Information:**

The online version contains supplementary material available at 10.1186/s12872-024-04048-x.

## Background

Vascular calcification (VC) is defined as the deposition of calcium-phosphate complexes in the cardiovascular system, which increases the morbidity and mortality of cardiovascular diseases [1–6]. VC was initially thought to be a passive process, but in fact it is an active and tightly regulated process with complex mechanisms [7, 8]. The differentiation of vascular smooth muscle cells (VSMCs) into osteoblast-like cells is considered to play a key role in the progression of VC [9, 10]. Therefore, it is important to explore signaling pathway to alleviate osteogenic differentiation in VSMCs, which can improve treatment options for VC. In recent years, nitrogen-containing bisphosphonate (N-BP) has been shown to have an inhibitory effect on the osteogenic differentiation and calcification in VSMCs [11, 12]. However, it is not clear how N-BP inhibits the osteogenic differentiation and calcification in VSMCs via specific regulatory mechanisms.

**Fig. 1:**
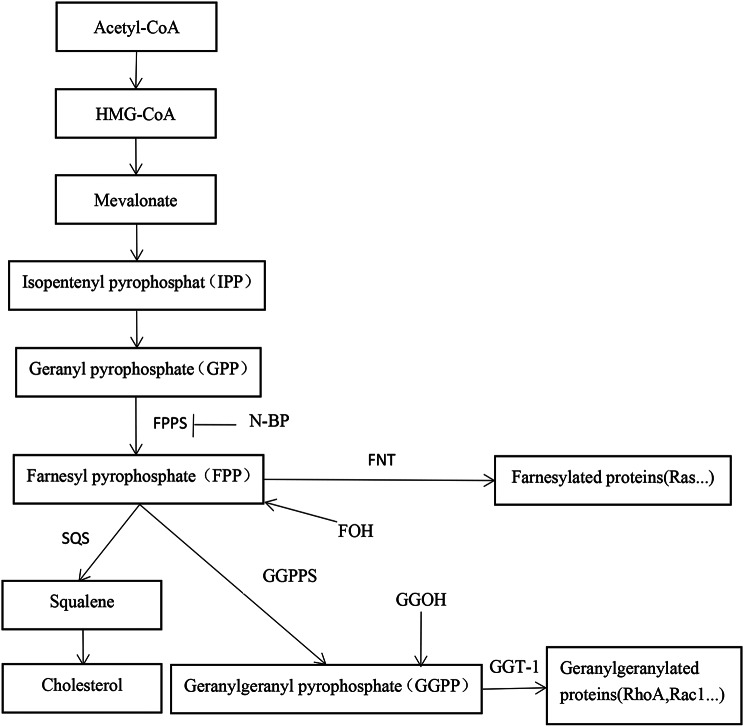
Schematic diagram of the mevalonate pathway. FPPS, farnesyl pyrophosphate synthase; SQS, squalene synthase; FNT, farnesyltransferase; GGT-1, geranylgeranyltransferase-1; GGPPS, geranylgeranyl pyrophosphate synthase; N-BPs, nitrogen-containing bisphosphonatesand; FOH, farnesol; GGOH, geranylgeraniol

**Fig. 2:**
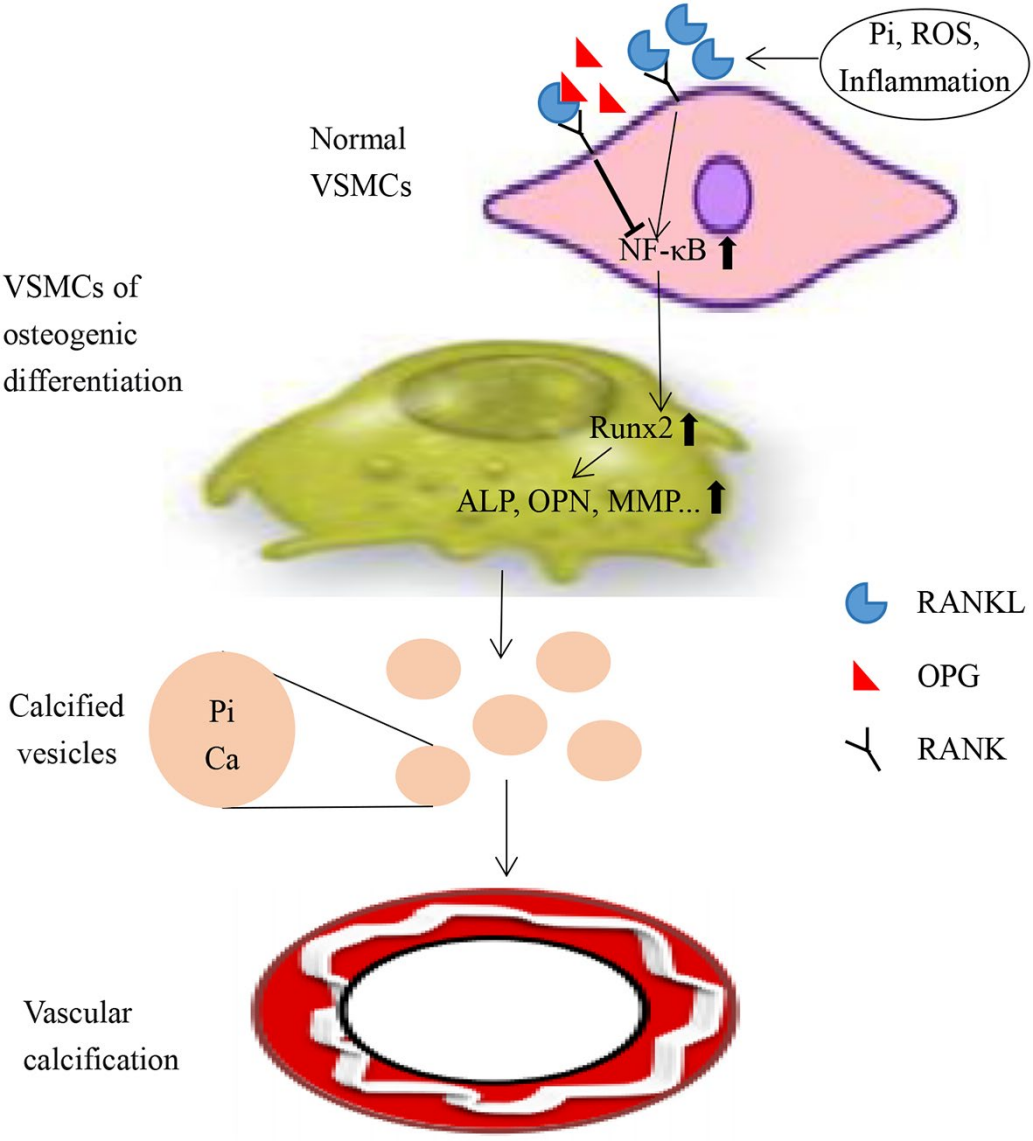
The role of RANKL/OPG in vascular calcification

N-BP, such as zoledronic acid(ZOL), is the drug for the treatment of osteoporosis. Both epidemiological and clinical studies have shown that patients with low bone mineral density are at significantly increased risk of VC [13, 14]. Some studies also suggested that the drugs that are effective on bone metabolism could also be effective on VC [13, 15]. N-BP has a high affinity for bone tissue and inhibit the activity of farnesate pyrophosphate synthetase(FPPS), leading to osteoclast apoptosis [16]. FPPS is an important enzyme in mevalonate metabolic pathway, so inhibition of FPPS by N-BP can block mevalonate pathway (Fig. 1) [13, 17]. In the study of Tsubaki, N-BP increased OPG and inhibited RANKL in mouse bone marrow stromal cell [18]. At the same time, this effect of N-BP could be reversed by adding downstream products of FPPS, such as farnesyl pyrophosphate (FPP), geranylgeranyl pyrophosphate (GGPP) [18]. The study of Pan and Viereck also showed that N-BP increased OPG and inhibited RANKL in the human osteoblast cells [19, 20]. However, in different cells, different concentrations of N-BP can regulate OPG and RANKL in different directions [21–24]. Regarding the mechanism of osteogenic differentiation and calcification in VSMCs, RANKL can promote the osteogenic differentiation and calcification in VSMCs via the RANKL/RANK signaling pathway. OPG can bind RANKL competitively to block the RANKL/RANK signaling pathway, which plays a protective role in the osteogenic differentiation and calcification in VSMCs (Fig. 2) (26,27). If N-BP can increase OPG and inhibit RANKL via inhibition of FPPS in VSMCs, it may alleviate osteogenic differentiation and calcification in VSMCs. At present, the clinical studies did not show the protective effect of N-BP on VC [25, 26]. The current experimental studies showed the protective effect of N-BP on VC, but the mechanism is unclear [27, 28]. Therefore, we intend to verify that N-BP induces enhancement of OPG expression and inhibition of RANKL expression via inhibition of FPPS to inhibit the osteogenic differentiation and calcification in VSMCs.

## Methods

### Cells culture and interventions

The VSMCs (Rat aortic thoracic smooth muscle cells, A7r5) were bought from Cell Bank, Chinese Academy of Sciences. The VSMCs were cultured in DMEM medium (Gibco, USA) containing 10% FBS (ExCell, China) at 37 °C in a 5% CO2 incubator.The culture medium was updated every 2–3 days.VSMCs less than 10 passages were used for experiments. To establish a model of osteogenic differentiation and calcification in VSMCs, the VSMCs were induced in DMEM medium containing 10 mM β-glycerophosphate (β-GP) (Sigma-Aldrich, USA) for 72 h. In some experiments, the VSMCs were preincubated for 2 h with 1 µM or 5 µM ZOL (MedChemExpress, USA), then VSMCs were co-cultured with or without 10 mM β-GP for 72 h. Furthermore, in some other experiments, the VSMCs were preincubated for 2 h with 5 µM ZOL, 30 µM farnesol (FOH)(Sigma-Aldrich, USA) plus 5 µM ZOL or 30 µM geranylgeraniol (GGOH)(Sigma-Aldrich, USA) plus 5 µM ZOL, then the VSMCs were co-cultured with or without 10 mM β-GP for 72 h. In the control group, the VSMCs were not given any intervention.

### Alizarin red S staining

Alizarin red S staining was performed to detect calcium deposition. The VSMCs were washed three times with Phosphate-Buffered Saline (PBS)(Beyotime, China), fixed in 4% paraformaldehyde for 30 min at room temperature, then stained with 1% Alizarin red S solution (Solarbio, China) for 5 min at room temperature. Subsequently, the VSMCs were washed with distilled water. The formation of calcified purple-red spots was quantified by microscopy(Olympus, Japan).

### Determination of calcium content

The calcium contents were determined by Calcium Assay Kit (Beyotime, China) according to the manufacturer’s instructions. 100–200 µl sample lysate was added to each well of the 6-well plate. The VSMCs were fully lysed and the supernatant was separated by centrifugation. The o-cresolphthalein complexone and detection buffer were mixed 1:1 to prepare the detection working solution for use. Then,50 µl sample and 150 µl detection working solution were added to each well of the 96-well plate and mixed well. The absorbance was assessed at 575 nm using an enzyme-labeled instrument. The total protein concentration was determined by BCA Protein Assay. The relative calcium content normalized to the protein concentration was expressed as µg/mg protein.

### Western blotting

Total protein was extracted from the VSMCs using RIPA lysis buffer (Beyotime, China) supplemented with protease inhibitor (Beyotime, China). The protein concentrations were detected using a BCA protein assay kit (Beyotime, China). Equal amounts of protein lysates were loaded and separated on a 10% SDS-PAGE gels and transferred onto polyvinylidene fluoride membranes (Beyotime, China). After blocking with 5% nonfat milk (diluted in Tris-buffered saline with Tween-20) for 2 h at room temperature, the membranes were incubated with primary antibodies at 4 °C overnight and incubated with secondary antibodies for 2 h at room temperature. The primary antibodies were as follows: Anti-RANKL (1:1000, Proteintech, China), Anti-OPG (1:1000, ABclonal, China), Anti-RUNX2 (1:1000, Proteintech, China), OPN (1:2000, Proteintech, China), GAPDH (1:2000, Proteintech, China). The secondary antibodies were as follows: HRP Goat Anti-Mouse IgG (1:5000, ABclonal, China) and HRP Goat Anti-Mouse IgG (1:5000, ABclonal, China). Proteins were detected by ECL chemiluminescence detection reagent (vazyme, China) and Amersham Imager 600 (GE Healthcare, UK). Western blotting results were quantitated using Image J software. Protein expression was normalized to GAPDH.

### Statistical analysis

All the data were continuous data and presented as mean ± SD. All results were obtained from 3 identical independent experiments. Differences between two groups were compared using Student’s t-test. The Student’s t-test was used to verify the induction of osteogenic differentiation and calcification model in VSMCs by β-GP. Differences among more than two groups were compared using one-way ANOVA. The one-way ANOVA was used to verify the mechanism and effect of N-BP on osteogenic differentiation and calcification in VSMCs. All statistical analyses were performed by use of SPSS 20.0 software. The graphs were plotted by GraphPad Prism 8.0 software. *P* < 0.05 was considered as statistically significant.

## Results

### Induction of osteogenic differentiation and calcification model in VSMCs by β-GP

**Fig. 3:**
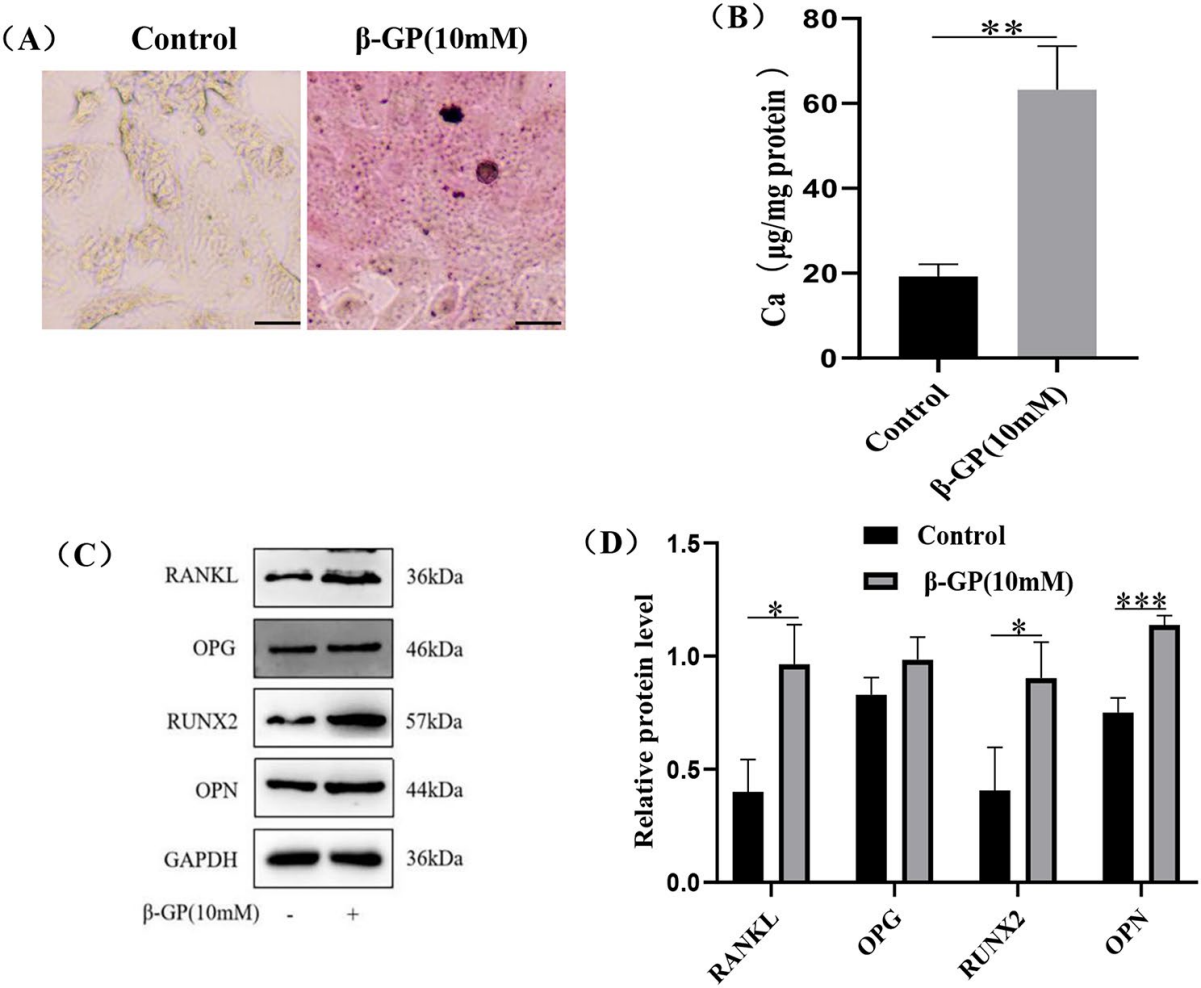
Induction of osteogenic differentiation and calcification model in VSMCs. VSMCs were treated with or without 10 mM β-GP for 72 h. (**A**) Alizarin Red S Staining was used to assess VSMCs calcification. Representative images showed VSMCs calcification with purple-red spots. Scale bar = 10 μm. (**B**) Calcium content was detected by Calcium Assay Kit. (**C**, **D**) Representative western blotting for RANKL, OPG and calcification-related proteins( Runx2 and OPN). Statistical significance was analyzed by the t-test (*p < 0.05, **p < 0.01, ***p < 0.001). The data is represented as mean ± SD (*n* = 3)

To establish osteogenic differentiation and calcification model in VSMCs, we stimulated VSMCs with 10mM β-GP for 72 h. The calcium deposition(purple-red spots)was induced in VSMCs at 72 h (Fig. 3A). The calcium contents in the β-GP group were also higher than that in control group (Fig. 3B, p < 0.01). The expression levels of osteogenic marker proteins Runx2 and OPN in the β-GP groupwere elevated in comparison to the control group (Fig. 3C, D, *p* < 0.05). Meanwhile, the expression levels of RANKL in the β-GP group were elevated in comparison to the control group (Fig. 3C, 3D, *p* < 0.05), but the expression levels of OPG in the β-GP group were not significantly higher than that in the control group (Fig. 3C, 3D, *p* > 0.05).

### Effect of N-BP on VSMCs calcification

**Fig. 4:**
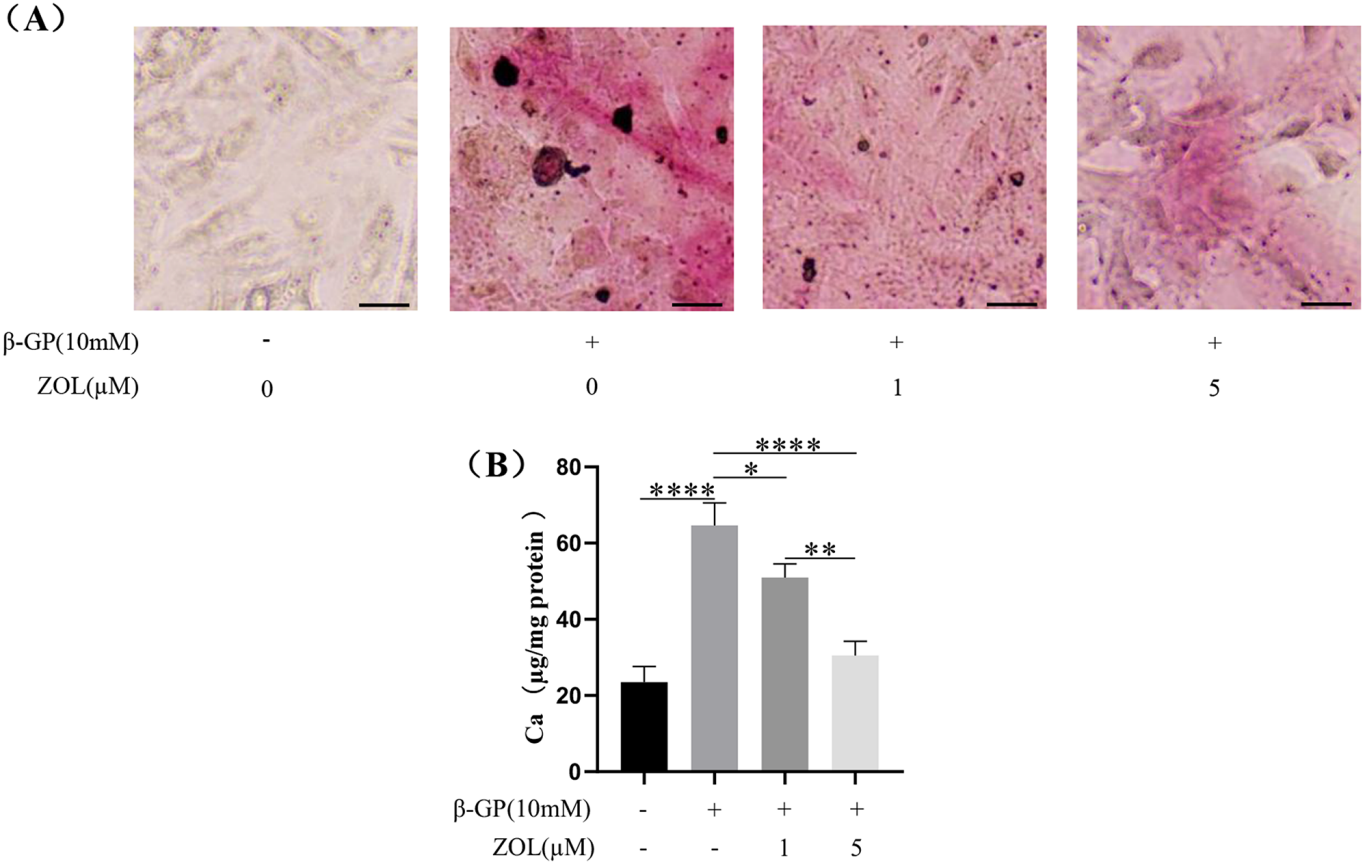
Effect of N-BP on VSMCs calcification. VSMCs were preincubated for 2 h with 1 µM or 5 µM ZOL, then VSMCs were co-cultured with or without 10 mM β-GP for 72 h. (**A**) Alizarin Red S Staining was used to assess VSMCs calcification. Representative images showed VSMCs calcification with purple-red spots. Scale bar = 10 μm. (**B**) Calcium content was detected by Calcium Assay Kit. Statistical significance was analyzed by one-way ANOVA (*p < 0.05, **p < 0.01, ****p < 0.0001). The data is represented as mean ± SD (*n* = 3)

ZOL (a type of N-BP) reduced the purple-red calcium deposition induced by β-GP in VSMCs at 72 h. Compared with 1µM ZOL, 5µM ZOL had a more obvious effect on reducing the purple-red calcium deposition in calcified VSMCs **(**Fig. 4A**)**. Meanwhile, ZOL reduced the calcium contents in calcified VSMCs at 72 h **(**Fig. 4B, p **< 0.05)**.Compared with 1µM ZOL, 5µM ZOL had a more obvious effect on reducing the calcium contents in calcified VSMCs **(**Fig. 4B, p **< 0.01)**.

### Effect of N-BP on RANKL, OPG and osteogenic marker proteins (Runx2 and OPN) expression in VSMCs of osteogenic differentiation and calcification

**Fig. 5:**
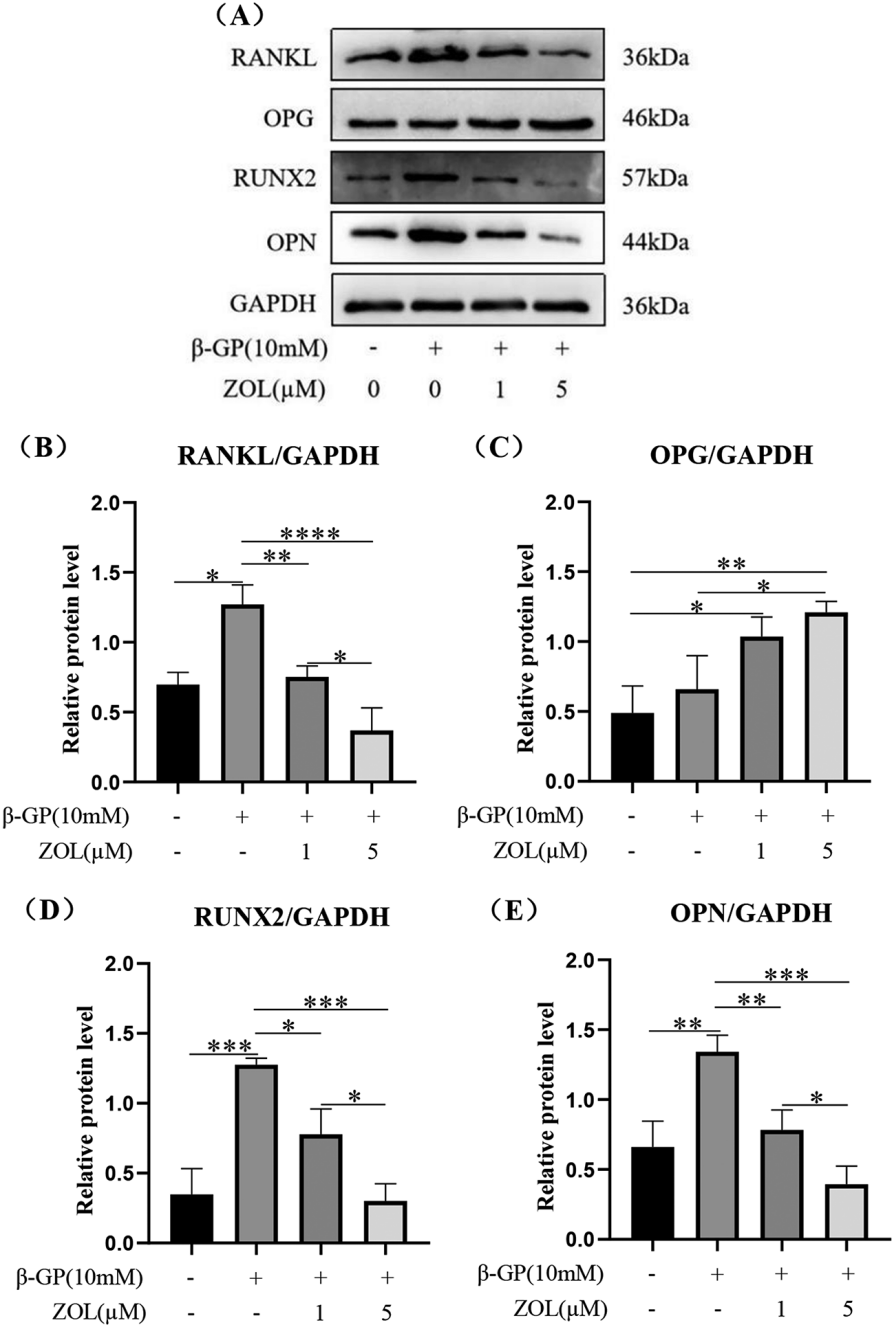
Effect of N-BP on RANKL, OPG and osteogenic marker proteins (Runx2 and OPN) expression in VSMCs of osteogenic differentiation and calcification. VSMCs were preincubated for 2 h with 1 µM or 5 µM ZOL, then VSMCs were co-cultured with or without 10 mM β-GP for 72 h. Statistical significance was analyzed by the one-way ANOVA (*p < 0.05, **p < 0.01, ***p < 0.001, ****p < 0.0001). The data is represented as mean ± SD (*n* = 3)

ZOL reduced the protein expression levels of RANKL in VSMCs undergoing osteogenic differentiation and calcification at 72 h (Fig. 5A, B, *p* < 0.05). Compared with 1µM ZOL, 5µM ZOL had a more obvious effect in reducing the protein expression levels of RANKL (Fig. 5A, B, *p* < 0.01). The protein expression levels of OPG in the β-GP group were slightly higher than that in the control group at 72 h, but the difference was not statistically significant (Fig. 5A, C, *p* > 0.05). Compared with the control group, ZOL plus β-GP group had the higher protein expression levels of OPG at 72 h (Figs. 5A, 3C, *p* < 0.05). Compared with the β-GP group, 1µM ZOL plus β-GP group increased the protein expression levels of OPG at 72 h, but the difference was not statistically significant (Fig. 5A, C, *p* > 0.05). Compared with the β-GP group, 5µM ZOL plus β-GP group had the higher protein expression levels of OPG at 72 h (Fig. 5A, C, *p* < 0.05). In addition, ZOL reduced the osteogenic marker proteins Runx2 and OPN in VSMCs undergoing osteogenic differentiation and calcification at 72 h (Fig. 5A, D. 5E, *p* < 0.05). Compared with 1µM ZOL, 5µM ZOL had a more obvious effect in reducing the protein expression levels of Runx2 and OPN (Fig. 5A, D. 5E, *p* < 0.05).

### Effect of N-BP on VSMCs calcification due to inhibition of FPPS

**Fig. 6:**
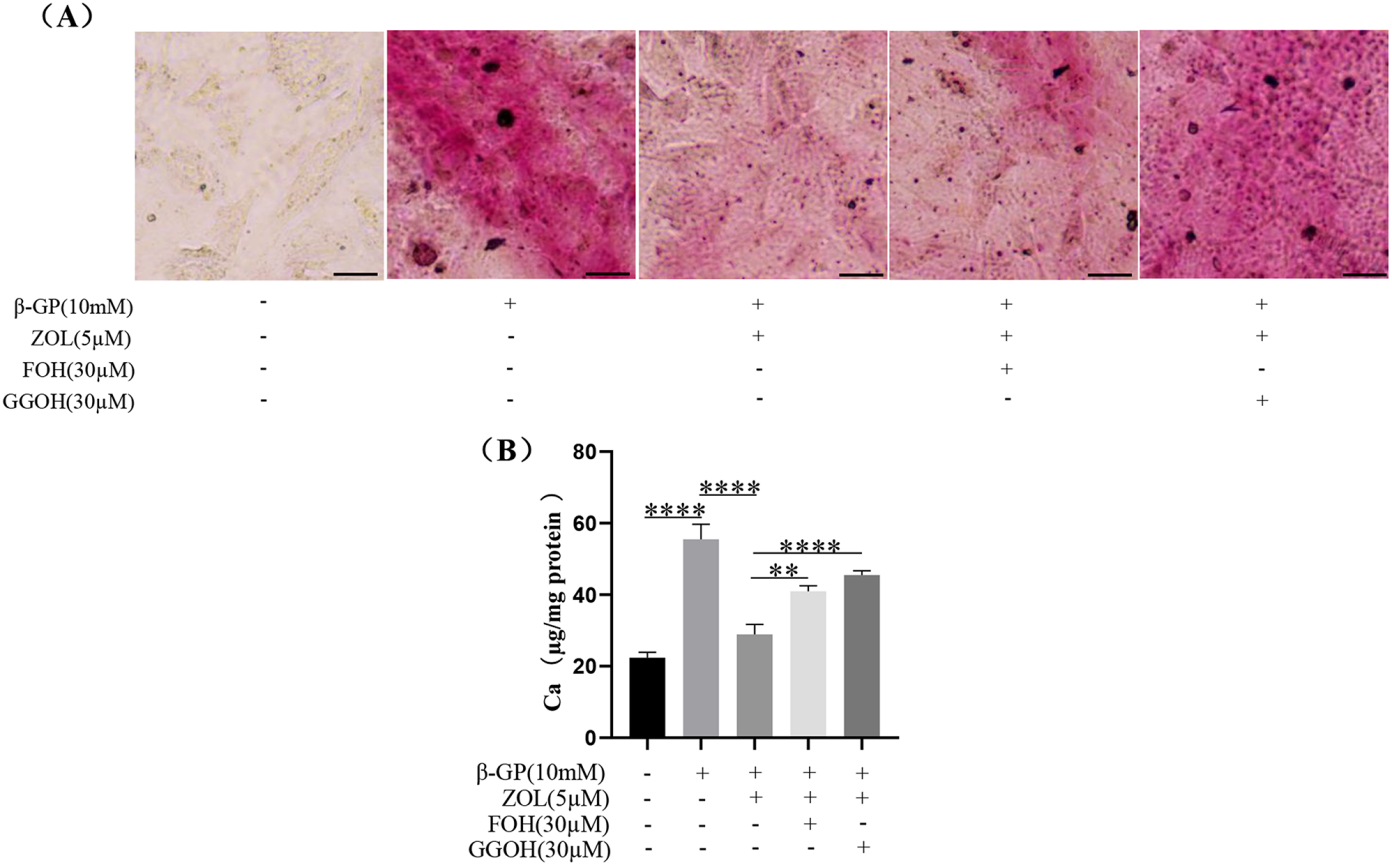
Effect of N-BP on VSMCs calcification due to inhibition of FPPS. VSMCs were preincubated for 2 h with 5 µM ZOL, 30 µM FOH plus 5 µM ZOL or 30 µM GGOH plus 5 µM ZOL, then VSMCs were co-cultured with or without 10 mM β-GP for 72 h. FOH and GGOH were the downstream products of FPPS in mevalonate pathway. (**A**) Alizarin Red S Staining was used to assess VSMCs calcification. Representative images showed VSMCs calcification with purple-red spots. Scale bar = 10 μm. (**B**) Calcium content was detected by Calcium Assay Kit. Statistical significance was analyzed by one-way ANOVA (*p < 0.05, **p < 0.01, ****p < 0.0001). The data is represented as mean ± SD (*n* = 3)

In order to reverse the inhibition effect of N-BP on VSMCs calcification due to inhibition of FPPS, we added the downstream products of FPPS in mevalonate pathway, such as FOH or GGOH. ZOL reduced the purple-red calcium deposition induced by β-GP in VSMCs at 72 h, which was reversed by FOH or GGOH **(**Fig. 6A**)**. Meanwhile, ZOL reduced the calcium contents in calcified VSMCs at 72 h **(**Fig. 6B, p **< 0.0001)**, which was also reversed by FOH or GGOH **(**Fig. 4B, p **< 0.01)**.

### Effect of N-BP on RANKL, OPG and osteogenic marker proteins( Runx2 and OPN) expression in VSMCs undergoing osteogenic differentiation and calcification due to inhibition of FPPS

**Fig. 7:**
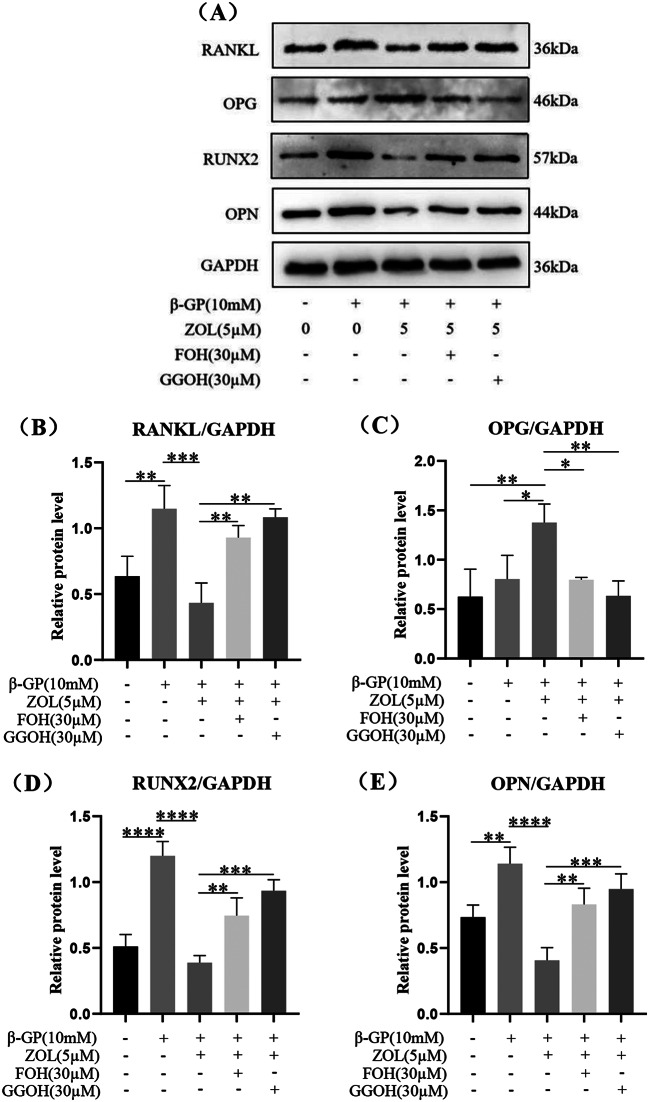
Effect of N-BP on RANKL, OPG and osteogenic marker proteins( Runx2 and OPN) expression in VSMCs of osteogenic differentiation and calcification due to inhibition of FPPS. VSMCs were preincubated for 2 h with 5 µM ZOL, 30 µM FOH plus 5 µM ZOL or 30 µM GGOH plus 5 µM ZOL, then VSMCs were co-cultured with or without 10 mM β-GP for 72 h. FOH and GGOH were the downstream products of FPPS in mevalonate pathway. Statistical significance was analyzed by the one-way ANOVA (*p < 0.01, **p < 0.01, ***p < 0.001, ****p < 0.0001). The data is represented as mean ± SD (*n* = 3)

In order to reverse the regulatory effect on RANKL, OPG and osteogenic marker proteins( Runx2 and OPN) expression in VSMCs of osteogenic differentiation and calcification due to inhibition of FPPS, we added the downstream products of FPPS in mevalonate pathway such as FOH or GGOH. 5µM ZOL induced enhancement of OPG protein expression and inhibition of RANKL protein expression in VSMCs undergoing osteogenic differentiation and calcification at 72 h (Fig. 7A, B, C, *p* < 0.001), which was reversed by FOH or GGOH (Fig. 7A, B, C, *p* < 0.05). In addition, 5µM ZOL reduced the osteogenic marker proteins Runx2 and OPN in VSMCs undergoing osteogenic differentiation and calcification at 72 h (Fig. 7A, D, E, *p* < 0.0001), which was reversed by FOH or GGOH (Fig. 7A, D, E, *p* < 0.01).

## Discussion

In this study, we investigated the effect and mechanism of N-BP on the osteogenic differentiation and calcification in VSMCs. In terms of the osteogenic differentiation and calcification in VSMCs **(**Figure 2**)**, previous studies showed that Runx2 was a key marker to drive this phenotypic differentiation. Runx2 is usually up-regulated during the osteogenic differentiation in VSMCs, promotes the expression of downstream osteogenic marker such as OPN, promotes the secretion of calcified vesicles, and finally leads to the deposition of calcium-phosphate complexes in the vascular wall [29, 30]. In adition, previous studies showed RANKL could promote the osteogenic differentiation and calcification via the RANKL/RANK signaling pathway in VSMCs, which also could up-regulate the expression of Runx2 and OPN [31, 32]. OPG can bind RANKL competitively to block the osteogenic differentiation and calcification in VSMCs [31, 32].

β-GP is often used to induce the osteogenic differentiation and calcification in VSMCs. As shown in Fig. 3 and 10mM β-GP could induce the osteogenic differentiation and calcification in VSMCs at 72 h, which was consistent with the result of Huqiang He [33]. Meanwhile, the expression of RANKL protein was up-regulated in VSMCs undergoing the osteogenic differentiation and calcification induced by β-GP, which was consistent with the result of Jinmi Lee and Liu [34, 35]. RANKL is a molecule associated with inflammation and previous study had found that expression of RANKL was increased in calcified arteries [36]. The expression of OPG protein was slight up-regulated in VSMCs undergoing osteogenic differentiation and calcification induced by β-GP, but the expression of OPG protein in the β-GP group had no statistical significance compared with that in the control group. In terms of the OPG expression during the osteogenic differentiation and calcification in VSMCs, the previous studies had no consensus. In the study of Huqiang He, the expression of OPG protein was up-regulated during the osteogenic differentiation and calcification induced by β-GP in VSMCs [33]. In the study of Yang Ho Kang, the expression of OPG protein was down-regulated slightly during the osteogenic differentiation and calcification induced by β-GP at 4 weeks in VSMCs [37]. It’s important to note that the expression of OPG protein was up-regulated slightly during the osteogenic differentiation and calcification induced by β-GP at 2 weeks in VSMCs in the study of Yang Ho Kang [37]. Previous study also found that patients with VC had elevated serum OPG [38]. Some scholars speculated that the expression of OPG in VSMCs was elevated as a defensive manner against VC for short-term stimulation in calcification medium [37, 38]. However, but the expression of OPG was down-regulated after long term stimulation with high phosphate, which could reduce the binding of OPG to RANKL and accelerate VC [38]. In our study, the VSMCs were only co-cultured with by β-GP for 72 h, so the stimulation of the VSMCs by the high phosphorus was short-term and the expression of OPG protein was up-regulated slightly.

In our study, ZOL, a kind of N-BP, inhibited the osteogenic differentiation and calcification in a concentration-dependent manner in VSMCs. The concentrations of ZOL we chosed were 1 and 5 µM, because previous study showed that a peak serum concentration of ZOL in human body following a 4 mg dose administration was only from 1 to 5 µM [39]. In our preliminary experiment, we found that the number of VSMCs co-cultured with 10–100 µM ZOL for 72 h was significantly reduced, so we did not choose ZOL > 5 µM to explore the effect on osteogenic differentiation and calcification in VSMCs. Meanwhile, we found ZOL increased the expression of OPG protein and decreased the expression of RANKL protein in VSMCs undergoing osteogenic differentiation and calcification, which could down-regulate the expression of osteogenic marker protein Runx2 and OPN to inhibit the osteogenic differentiation and calcification in VSMCs. The previous studies showed that other kind of N-BP could also inhibit the osteogenic differentiation and calcification in VSMCs [11, 12], but the researchers did not find that N-BP inhibited this phenotypic differentiation via enhancement of OPG expression and inhibition of RANKL expression. The previous study also found N-BP induced enhancement of OPG expression and inhibition of RANKL expression in mouse bone marrow stromal cells [18]. However, some studies found that the regulatory effects of different concentrations of N-BP on OPG and RANKL in different cells are unclear [21–24]. Our experiment confirmed that 1–5 µM N-BP could increased the expression of OPG and decreased the expression of RANKL in VSMCs, which could inhibit osteogenic differentiation and calcification in VSMCs finally. N-BP itself has an inhibitory effect on FPPS.Thus, by the addition of FPPS downstream products such as FOH or GGOH in mevalonate pathway, we further verified in reverse that N-BP regulated OPG and RANKL via inhibiting FPPS. The previous study showed that the involvement of FPPS downstream products in prenylation of small GTPases (Ras and Rho) was clarified and these mall GTPases might play a major role in regulated OPG and RANKL [18]. In addition, most clinical studies found that N-BP did not inhibit vascular calcification in human [13, 40], which might be because the serum concentration of N-BP cannot be maintained consistently at 1–5 μm.

There were some limitations in our study. Firstly, this study was conducted in vitro. We still need to conduct experiments in vivo to more fully verify protective role and mechanism of N-BP on VC. Secondly, in this experiment, by adding downstream products of FPPS, N-BP was reversely verified to regulate RANKL and OPG by inhibiting FPPS. We hope to use more direct methods to explore the regulation of OPG and RANKL by FPPS in VSMCs in subsequent studies.

In conclusion, our findings demonstrated the protective role and mechanism of N-BP on the osteogenic differentiation and calcification in VSMCs. N-BP could induce enhancement of OPG expression and inhibition of RANKL expression via inhibition of FPPS, which could inhibit the osteogenic differentiation and calcification in VSMCs. Although the current clinical study did not show the protective effect of N-BP on VC, our experiment found that the regulation of N-BP on RANKL and OPG through inhibiting FPPS had reference value for future clinical treatment of VC. Perhaps, in the future, we can explore the treatment of VC in terms of N-BP drug dose, N-BP drug affinity to VSMCs, and FPPS enzyme downstream products.

## Electronic supplementary material

Below is the link to the electronic supplementary material.

Supplementary Material 1

## Data Availability

Data is provided within the manuscript or supplementary information files.

## Abbreviations

VC: Vascular calcification
VSMCs: Vascular smooth muscle cells
N-BP: Nitrogen-containing bisphosphonate
ZOL: Zoledronic acid
FPPS: Farnesate pyrophosphate synthetase
FPP: Farnesyl pyrophosphate
GGPP: Geranylgeranyl pyrophosphate
FOH: Farnesol
GGOH: Geranylgeraniol
β-GP: β-glycerophosphate
