# Supplementary material for: Nitrogen-containing bisphosphonate induces enhancement of OPG expression and inhibition of RANKL expression via inhibition of farnesyl pyrophosphate synthase to inhibit the osteogenic differentiation and calcification in vascular smooth muscle cells
Source: BMC Cardiovasc Disord. 2024 Sep 17;24:494. doi: 10.1186/s12872-024-04048-x (PMC11406803; doi:10.1186/s12872-024-04048-x)
Supplement: Supplementary file 1 — Supplementary Material 1 [file 12872_2024_4048_MOESM1_ESM.docx]

**Supplementary figure**

**Figure 3.** Induction of osteogenic differentiation and calcification model in VSMCs.Induction of osteogenic differentiation and calcification model in VSMCs. VSMCs were treated with or without 10 mM β-GP for 72 h.

(B) Calcium content was detected by Calcium Assay Kit. (C,D) Representative western blotting for RANKL, OPG and osteogenic marker proteins( Runx2 and OPN). Statistical significance was analyzed by the t-test (*p < 0.01, **p < 0.01, ***p < 0.001). The data is represented as mean ± SD (n = 3).

**Figure 3A.** Alizarin Red S Staining was used to assess VSMCs calcification. Representative images showed VSMCs calcification with purple-red spots. Scale bar = 10 µm.

、


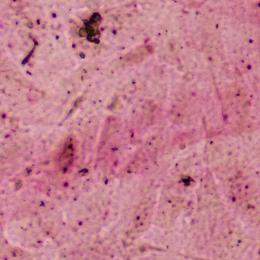

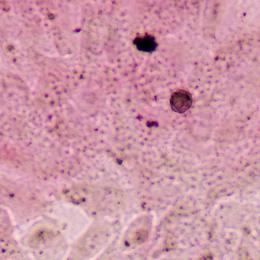

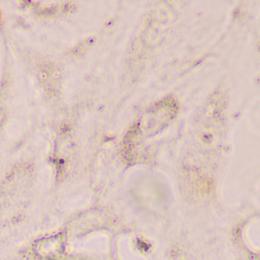

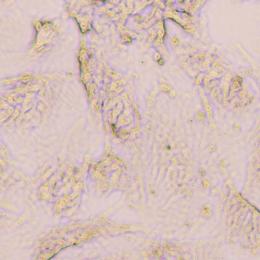

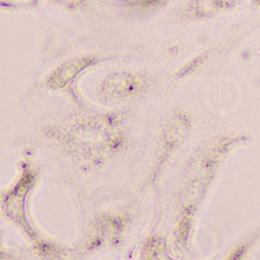


**Control**

**β-GP(10mM)**


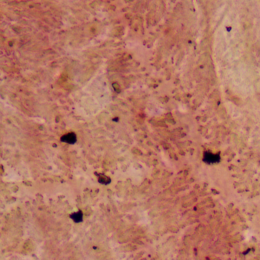


**Figure 3C.** Representative western blotting for RANKL, OPG and osteogenic marker proteins( Runx2 and OPN)

GAPDH

RANKL

OPG

RUNX2

OPN

**Figure 3C (1)**


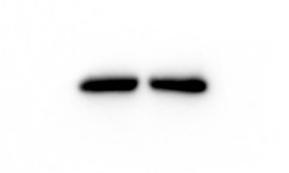

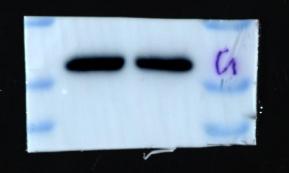


40kDa

35kDa


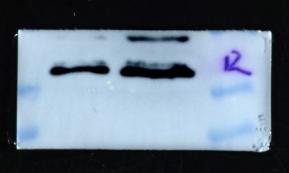


40kDa

35kDa


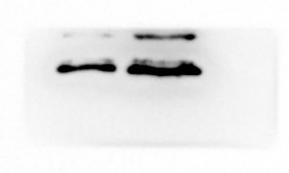

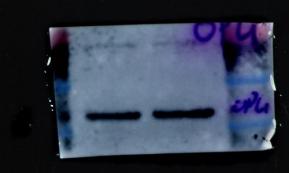


55kDa

40kDa


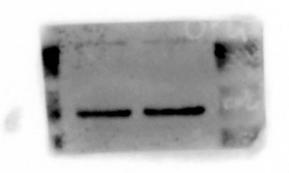

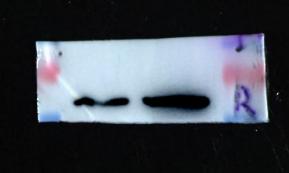


70kDa

55kDa


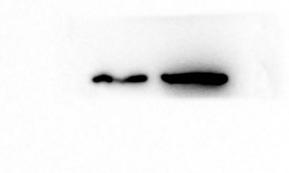

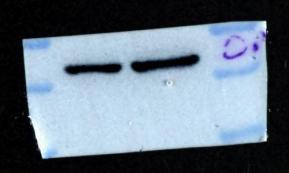


55kDa

40kDa


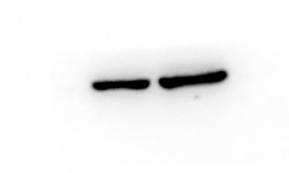


β-GP(10mM)

-

+

-

+

GAPDH

RANKL

OPG

RUNX2

OPN

**Figure 3C (2)**


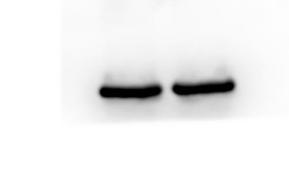

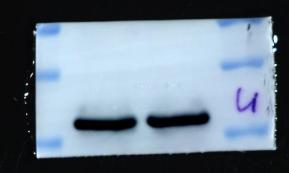


40kDa

35kDa


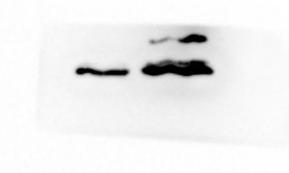

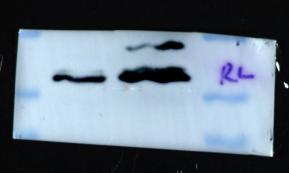


40kDa

35kDa


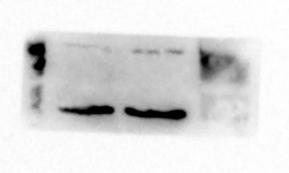

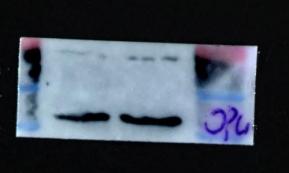


55kDa

40kDa


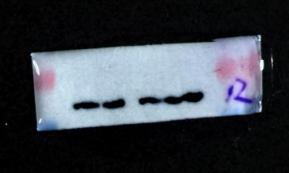


70kDa

55kDa


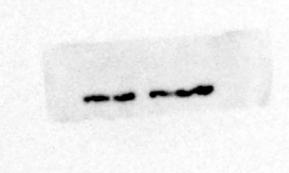

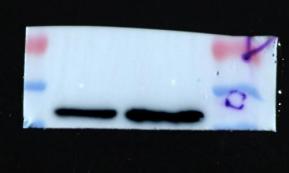


55kDa

40kDa


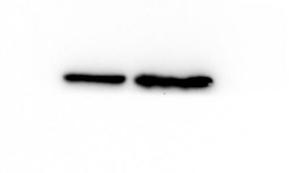


β-GP(10mM)

-

+

-

+

GAPDH

RANKL

OPG

RUNX2

OPN

**Figure 3C (3)**


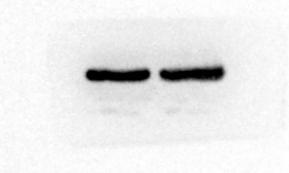

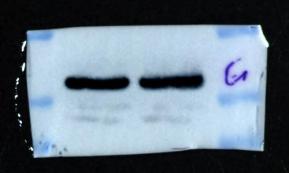


40kDa

35kDa


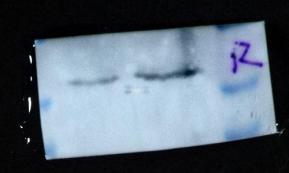


40kDa

35kDa


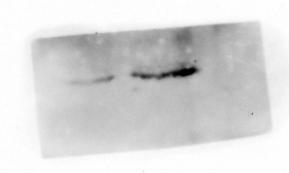

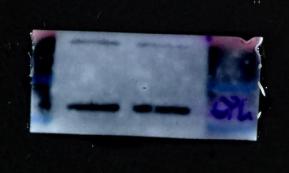


55kDa

40kDa


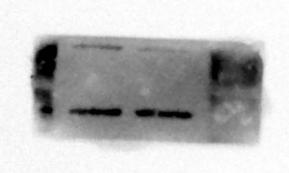

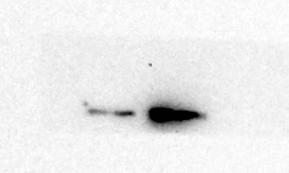

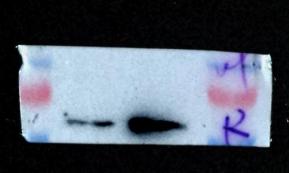


70kDa

55kDa


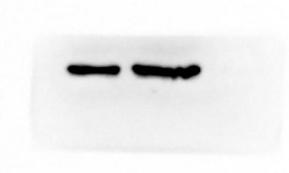

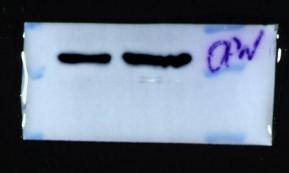


55kDa

40kDa

β-GP(10mM)

-

+

-

+

**Figure 4A** **.** Effect of N-BP on VSMCs calcification. VSMCs were preincubated for 2 h with 1 µM or 5 µM ZOL ,then VSMCs were co-cultured with or without 10 mM β-GP for 72 h. Alizarin Red S Staining was used to assess VSMCs calcification. Representative images showed VSMCs calcification with purple-red spots. Scale bar = 10 µm.


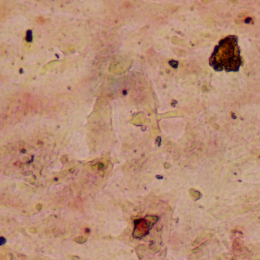

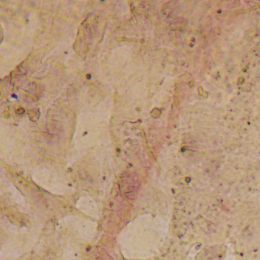

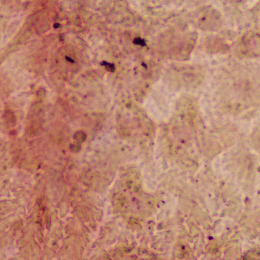

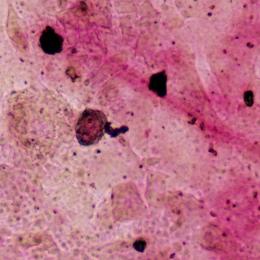

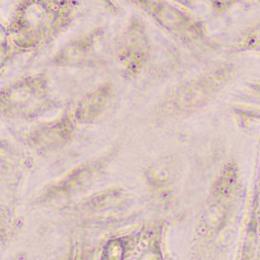

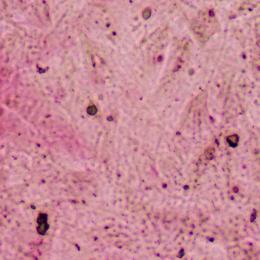

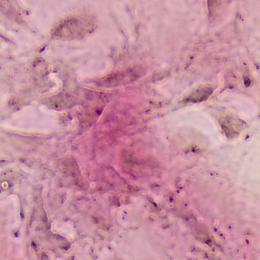

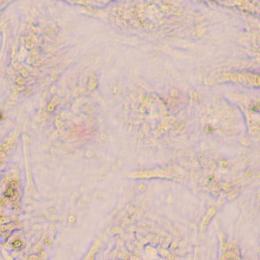

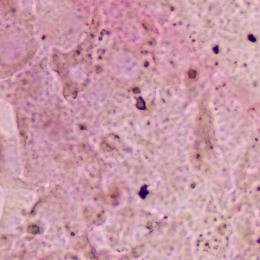

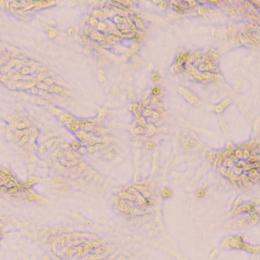

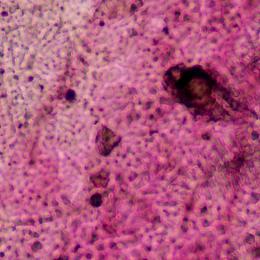

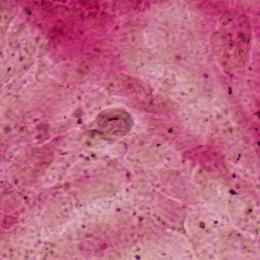


β-GP(10mM)

-

+

ZOL(µM)

0

0

+

1

+

5

**Figure 5A.** Effect of N-BP on RANKL, OPG and osteogenic marker proteins( Runx2 and OPN) expression in VSMCs of osteogenic differentiation and calcification. VSMCs were preincubated for 2 h with 1 µM or 5 µM ZOL ,then VSMCs were co-cultured with or without 10 mM β-GP for 72 h.

GAPDH

RANKL

OPG

RUNX2

OPN

**Figure 5A (1)**


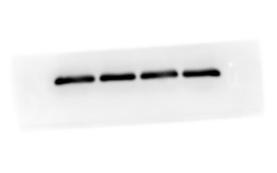

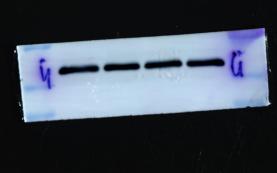


40kDa

35kDa


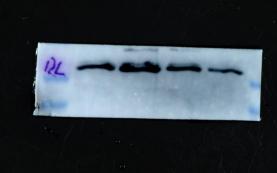


40kDa

35kDa


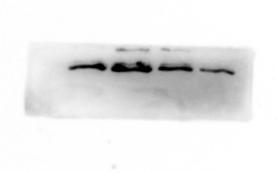

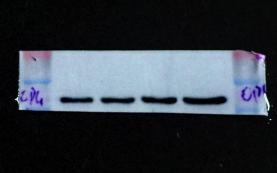


55kDa

40kDa


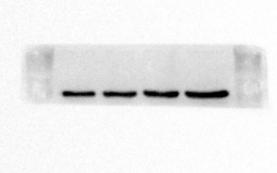

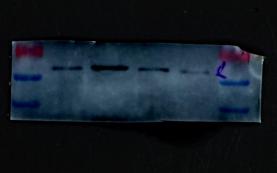


70kDa

55kDa


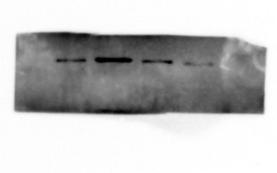

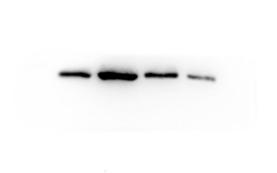

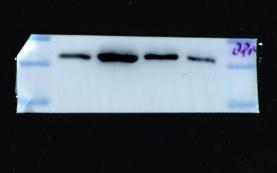


55kDa

40kDa

β-GP(10mM)

-

+

ZOL(µM)

0

0

+

1

+

5

-

+

0

0

+

1

+

5

GAPDH

RANKL

OPG

RUNX2

OPN

**Figure 5A (2)**

4组第2分组


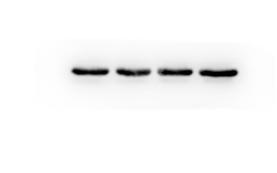

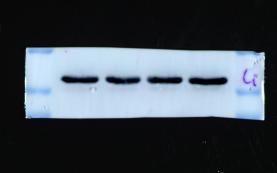


40kDa

35kDa


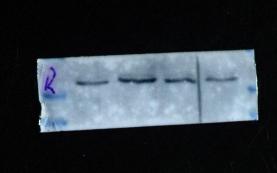


40kDa

35kDa


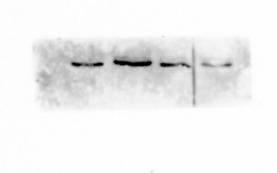

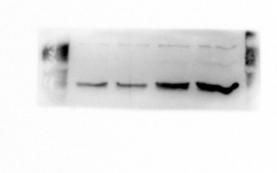

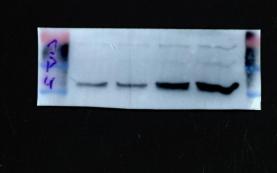


55kDa

40kDa


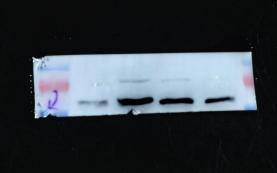


70kDa

55kDa


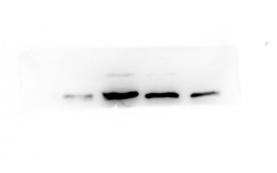

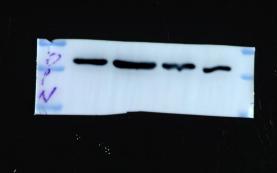


55kDa

40kDa


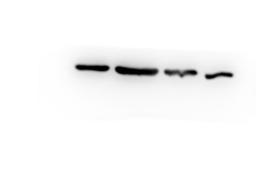


β-GP(10mM)

-

+

ZOL(µM)

0

0

+

1

+

5

-

+

0

0

+

1

+

5

GAPDH

RANKL

OPG

RUNX2

OPN

**Figure 5A (3)**


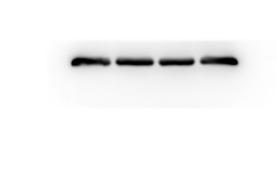

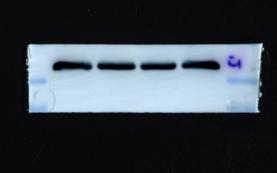


40kDa

35kDa


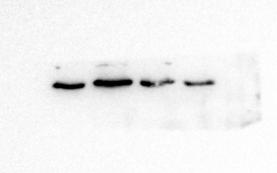

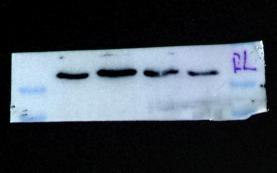


40kDa

35kDa


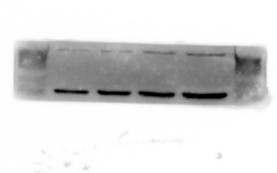

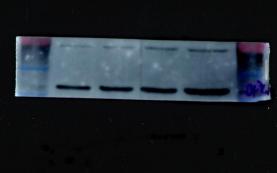


55kDa

40kDa


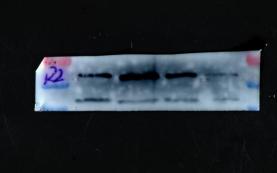


70kDa

55kDa


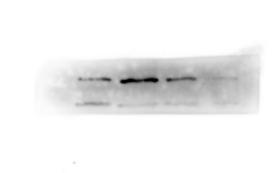

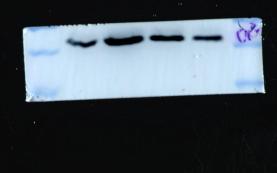


55kDa

40kDa


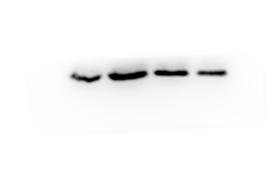


β-GP(10mM)

-

+

ZOL(µM)

0

0

+

1

+

5

-

+

0

0

+

1

+

5

**Figure 6A.** Effect of N-BP on VSMCs calcification due to inhibition of FPPS. VSMCs were preincubated for 2 h with 5 µM ZOL, 30 µM FOH plus 5 µM ZOL or 30 µM GGOH plus 5 µM ZOL, then VSMCs were co-cultured with or without 10 mM β-GP for 72 h. FOH and GGOH were the downstream products of FPPS in mevalonate pathway . Alizarin Red S Staining was used to assess VSMCs calcification. Representative images showed VSMCs calcification with purple-red spots. Scale bar = 10 µm.


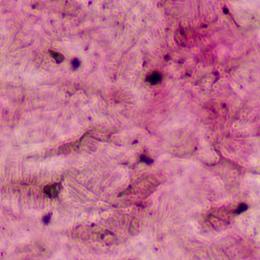

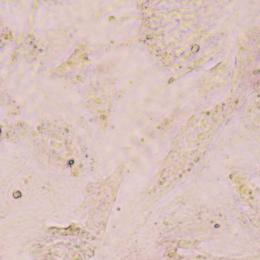

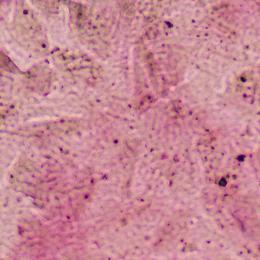

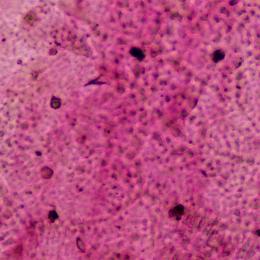

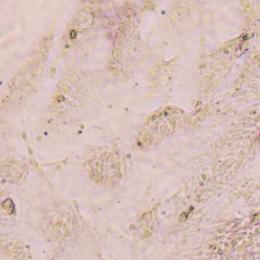

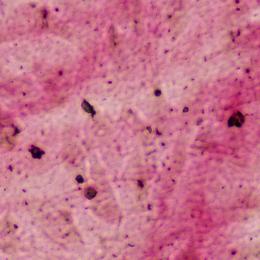

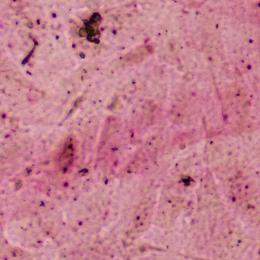

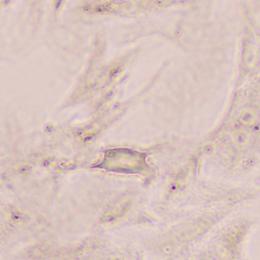

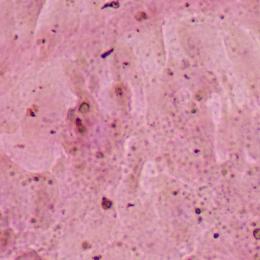

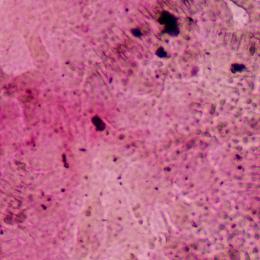

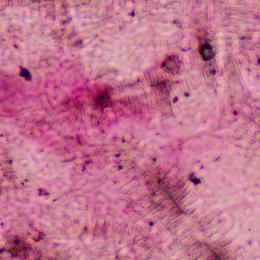

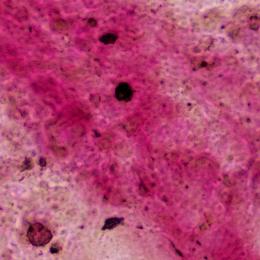

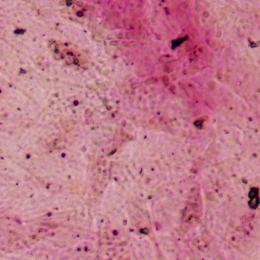

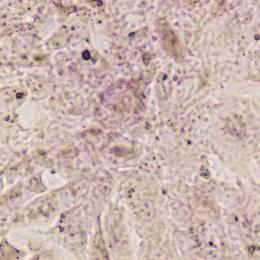

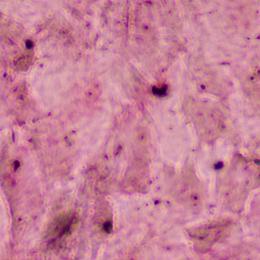


β-GP(10mM)

ZOL(5µM)

FOH(30µM)

GGOH(30µM)

-

-

-

-

+

+

+

-

+

+

-

-

+

-

-

-

+

+

-

+

**Figure 7A.** Effect of N-BP on RANKL, OPG and osteogenic marker proteins( Runx2 and OPN) expression in VSMCs of osteogenic differentiation and calcification due to inhibition of FPPS. VSMCs were preincubated for 2 h with 5 µM ZOL, 30 µM FOH plus 5 µM ZOL or 30 µM GGOH plus 5 µM ZOL, then VSMCs were co-cultured with or without 10 mM β-GP for 72 h. FOH and GGOH were the downstream products of FPPS in mevalonate pathway.

GAPDH

RANKL

OPG

RUNX2

OPN

**Figure 7A (1)**


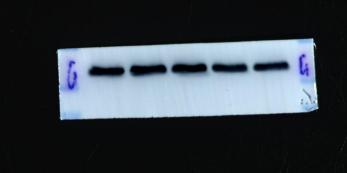


40kDa

35kDa


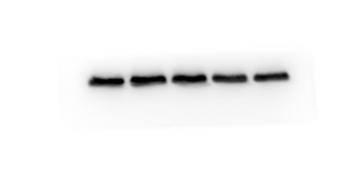

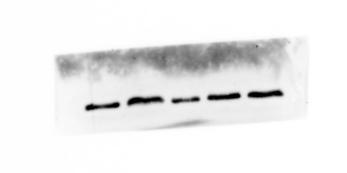

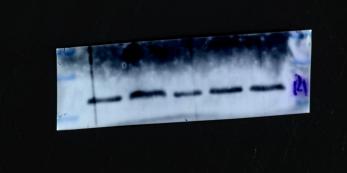


40kDa

35kDa


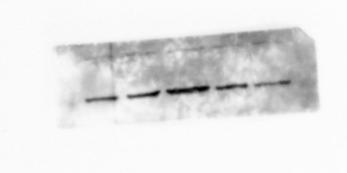

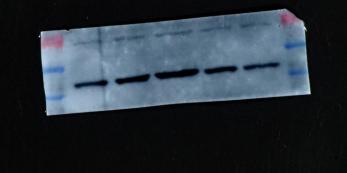


55kDa

40kDa


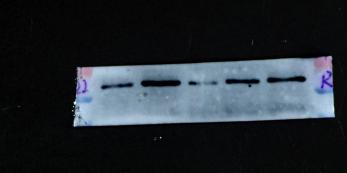


70kDa

55kDa

55kDa

40kDa

β-GP(10mM)

ZOL(5µM)

FOH(30µM)

GGOH(30µM)

-

-

-

-

+

+

+

-

+

+

-

-

+

-

-

-

+

+

-

+

-

-

-

-

+

+

+

-

+

+

-

-

+

-

-

-

+

+

-

+

GAPDH

RANKL

OPG

RUNX2

OPN

**Figure 7A (2)**

40kDa

35kDa

40kDa

35kDa

55kDa

40kDa

70kDa

55kDa

55kDa

40kDa

β-GP(10mM)

ZOL(5µM)

FOH(30µM)

GGOH(30µM)

-

-

-

-

+

+

+

-

+

+

-

-

+

-

-

-

+

+

-

+

-

-

-

-

+

+

+

-

+

+

-

-

+

-

-

-

+

+

-

+

GAPDH

RANKL

OPG

RUNX2

OPN

**Figure 7A (3)**

40kDa

35kDa

40kDa

35kDa

55kDa

40kDa

70kDa

55kDa

70kDa

55kDa

β-GP(10mM)

ZOL(5µM)

FOH(30µM)

GGOH(30µM)

-

-

-

-

+

+

+

-

+

+

-

-

+

-

-

-

+

+

-

+

-

-

-

-

+

+

+

-

+

+

-

-

+

-

-

-

+

+

-

+
